# End-to-Side Ileo-Sigmoid Anastomosis and Late Onset Gangrenous Diverticulitis: A Case Report

**DOI:** 10.7759/cureus.43441

**Published:** 2023-08-13

**Authors:** Mithilesh K Sinha, Shantanu Sahu, Upendra Hansda, Amarendra Kumar

**Affiliations:** 1 General Surgery, All India Institute of Medical Sciences, Bhubaneswar, Bhubaneswar, IND; 2 Trauma and Emergency, All India Institute of Medical Sciences, Bhubaneswar, Bhubaneswar, IND

**Keywords:** gangrenous diverticulitis, colonic diverticula, case report, bowel anastomosis, blind loop syndrome

## Abstract

End-to-side ileo-sigmoid anastomosis converts the proximal colon into a blind intestinal segment which is excised during the surgery. If we do not resect the proximal colon, it is expected to behave like a colonic diverticulum, but direct evidence of this assumption is lacking. During an exploratory laparotomy, we detected an end-to-side ileo-sigmoid anastomosis and found that the proximal colon was gangrenous. The patient passed away during the postoperative period, yet their remarkably long period of symptom-free survival remained intriguing.

## Introduction

End-to-side bowel anastomosis is executed in various surgical contexts, such as bariatric procedures like one-anastomosis gastric bypass and end-to-side jejunostomy, as well as after distal gastrectomy (Bilroth II procedure) and total gastrectomy [1]. In all these surgeries, the side limb of the bowel proximal to the anastomosis serves as a conduit for transporting bile and pancreatic juices. However, when a similar anastomosis is performed elsewhere in the intestinal tract, the side limb becomes non-functional and is therefore excised. In cases involving pathology or obstruction in the right side of the colon, palliation is occasionally achieved with a side-to-side ileo-colic anastomosis [2]. However, even in such circumstances, the literature lacks information on end-to-side ileo-sigmoid or ileo-colic anastomosis without resection of the redundant side limb of the bowel. Besides the initial risk of stump leakage during the postoperative period, our understanding of the late complications associated with this surgical procedure remains limited.

## Case presentation

A 75-year-old man was brought to the surgical emergency with abdominal pain and constipation for three days. The initial symptom was abdominal discomfort followed by continuous and severe pain for the past day. The patient vomited twice in the past day, which was non-bilious and contained food particles. There was a history of abdominal surgery 25 years back, but the patient could not detail it. His heart rate was 118 beats per minute, BP 160/110 mm Hg, and SPO2 98% on room air. On local examination, the abdomen was distended, and an 8 cm long right paramedian scar was on it. It was tender on palpation, and the bowel sounds were absent on auscultation. The rectum was empty on digital rectal examination.

**Figure 1:**
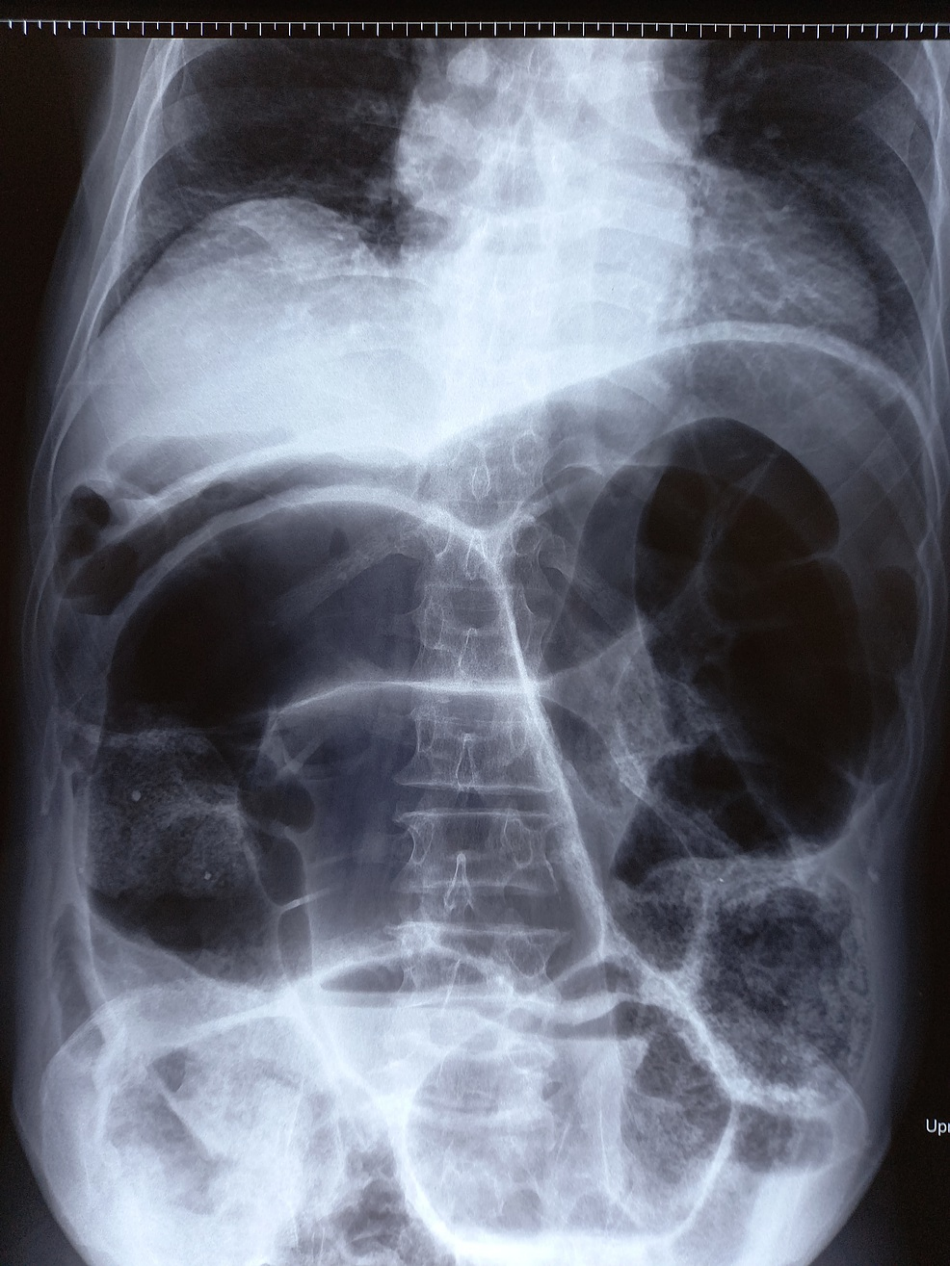
X-ray of the abdomen showing a distended large bowel.

An abdominal X-ray in supine posture was performed, and it showed significant gaseous distension of the large bowel (Figure 1).

Routine hemogram, serum electrolytes, blood urea, serum creatinine, serum amylase, and lipase and liver function tests were normal. We suspected an adhesive bowel obstruction with strangulation in this case. In this case, we also suspected large gut volvulus and decided on an exploratory laparotomy.

**Figure 2:**
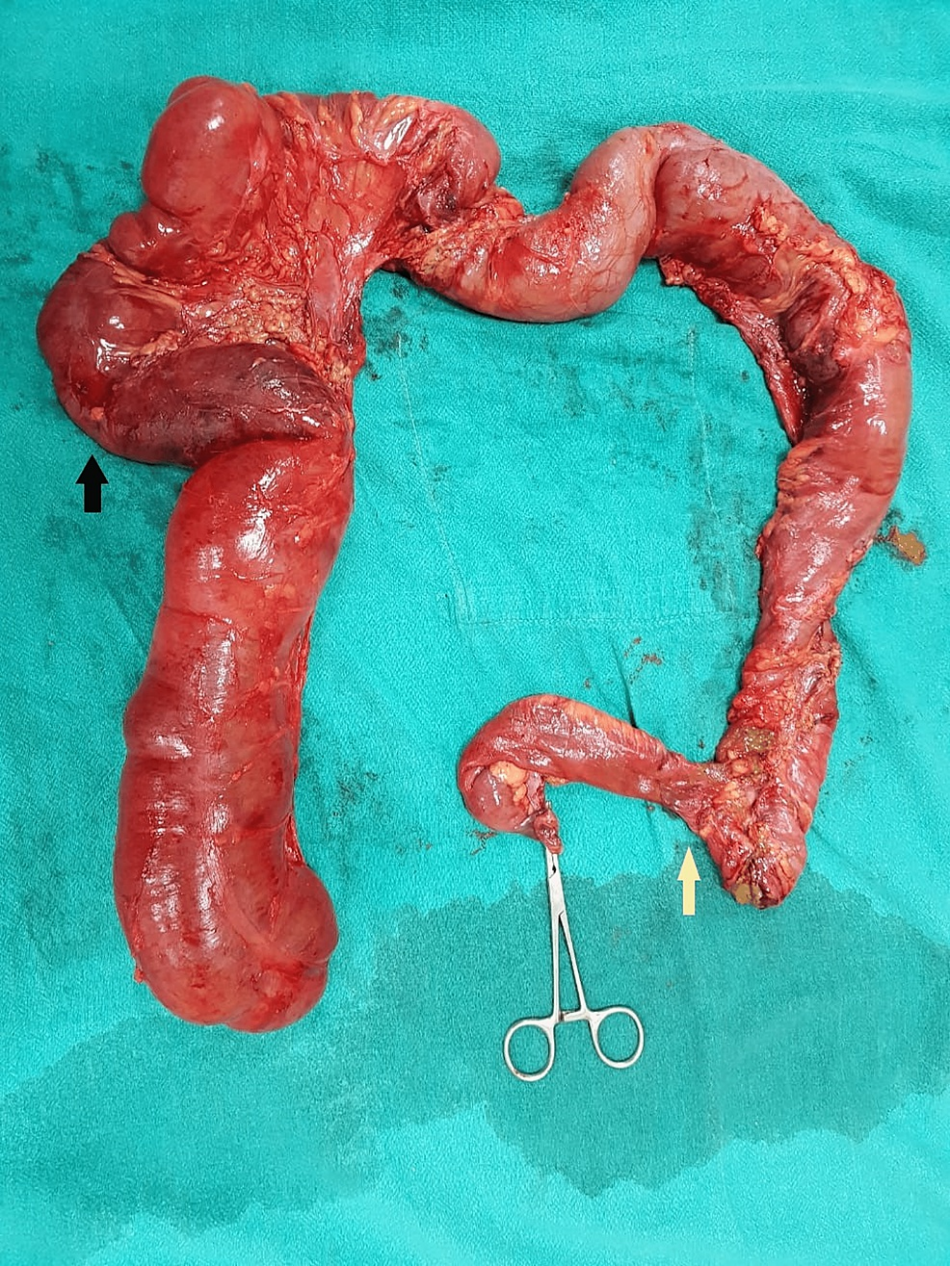
The resected specimen displays an end-to-side ileo-sigmoid anastomosis (indicated by the yellow arrow) and the enclosed segment of the colon proximal to it. Additionally, a patchy area of gangrene is evident near the hepatic flexure (marked by the black arrow).

An exploratory laparotomy through a midline incision was performed. A careful blunt and sharp dissection was performed to delineate the bowel anatomy. We found a distended and gangrenous ascending, transverse and descending colon with a distal end-to-side ileo-sigmoid anastomosis. The caecum was a blind tube, and the vermiform appendix was missing at its base (Figure 2).

We conducted a resection of the entire blind loop of the colon and subsequently performed Hartman's procedure by closing the bowel at the distal third of the sigmoid colon (Video 1).

**Video 1:** Video of the resected specimen showing ileo-sigmoid anastomosis, gangrenous regions of diverticula, and absence of vermiform appendix at the base of the caecum.

During the postoperative period, the patient remained intubated. They passed away due to sepsis and multi-organ dysfunction nearly 18 hours following the surgery.

## Discussion

A colonic diverticulum is an abnormal outpouching that often affects the ageing population, particularly on the left side of the colon. It may be present in 60% of individuals aged 70 and above, but it is mostly asymptomatic [3]. They are seldom larger than 4 cm; however, when they do grow beyond this size, they are referred to as giant colonic diverticula. These enormous diverticula can increase in size up to 9 cm, with the most extreme cases even attaining a diameter of 25 cm. The most common symptom associated with colonic diverticula is abdominal pain, present in nearly two-thirds of the patients. Other less common symptoms include constipation, abdominal distension, abdominal mass, and altered bowel habits. Although asymptomatic presentation has also been reported, it is less frequent. On physical examination, abdominal swelling that may be tender can be found [4].

The giant colonic diverticula is classified into three types. Type I diverticula, also known as pseudo-diverticula, are lined by remnants of mucosa or muscularis propria. Type II constitutes inflammatory diverticula, formed by persistent communication between a colonic perforation and a walled-off collection, and these are lined by fibrous scar tissue. Type III represents true giant diverticula, encompassing all three layers and often developing due to congenital anomalies. All forms of diverticula tend to progressively enlarge due to distension caused by trapped air and stool. Iatrogenic or surgically created diverticula are classified as true Type III diverticula, and our understanding of these cases remains limited [5]. In our specific case, the end-to-side ileo-sigmoid anastomosis transformed the initial 110 cm of the colon into a blind segment, similar to an abnormal sac originating from the sigmoid colon, resembling a Type III diverticulum. Intriguingly, our patient displayed no symptoms indicative of progressive distension or enlargement of this segment until experiencing acute abdominal pain leading to admission.

A giant colonic diverticulum as a result of faulty surgical technique is rare. We found one case report about the same in the literature [5]. Here the diverticula developed after the closure of the colostomy. It was detected 15 years after the surgery and was 14.5 cm in diameter. The patient had left lower abdominal fullness, occasional discomfort, and constipation for more than one year. The computed tomography (CT) scan of the abdomen and pelvis confirmed the diagnosis. This case was similar to ours as it presented very late after the surgery. However, it differed in that the complaints were chronic, the diverticulum size was smaller, and a side-to-side anastomosis was performed.

The presence of an intestinal blind loop can lead to blind loop syndrome or stagnant loop syndrome. This syndrome occurs due to the stagnation of contents, which promotes excessive growth of intestinal microflora. The composition of the microflora also changes, disrupting the digestive and absorptive functions of the intestine. This syndrome is associated with malabsorption of fat and deficiencies in essential nutrients such as vitamin B12, folate, and iron. Symptoms experienced by patients include loss of appetite, flatulence, steatorrhea (fatty stools), and weight loss. It's worth noting that the stasis of contents or the presence of diverticula in the colon may not necessarily lead to blind loop syndrome, as the microflora in the colon differs from that in the small bowel. Additionally, the movement of contents in the colon is inherently slower compared to the small bowel. The patient, in our case, survived for two and half decades and was not suffering from any deficiency syndrome. They probably had no appetite and bowel movement-related issues [6].

Colonic diverticula can be identified through a plain X-ray of the abdomen, where they manifest as smooth-walled, air-filled, rounded, or oval structures, or by using abdominal ultrasonography [7]. CT scan of the abdomen and pelvis is needed to confirm the diagnosis. Barium enema studies are of historical importance and are not recommended. During acute diverticulitis, sigmoidoscopy or colonoscopy should be avoided to lower the risk of perforation [8]. The treatment of colonic diverticulum needs individualization. A small and asymptomatic diverticulum needs no intervention. Once it becomes symptomatic, we can manage them with antibiotics. Patients with frequent symptoms affecting the quality of life or those experiencing complications need surgical intervention. Aspiration under radiological guidance is recommended for localized abscesses. An exploratory laparotomy is performed when patients present with generalized peritonitis [8].

The giant colonic diverticula are mostly symptomatic and often need surgical correction. Interestingly, the blind colonic segment following ileo-sigmoid anastomosis remained nearly asymptomatic for 25 years. The blind colonic segment was larger than any reported natural diverticula. The sudden development of gangrene in this segment is a complication that also affects colonic diverticula. We are reporting this case as it describes the long-term outcome of end-to-side ileo-sigmoid anastomosis without resection of the colon, a procedure not advisable in surgery under normal circumstances.

## Conclusions

It is advisable to revise a surgically created giant colonic diverticulum to prevent delayed complications. Surgically created colonic diverticula may sometimes remain asymptomatic for an extended period and are typically not associated with blind loop syndrome.
